# Pressure Induced Spin Crossover and Magnetic Properties of Multiferroic Ba_3_NbFe_3_Si_2_O_14_

**DOI:** 10.3390/molecules25173808

**Published:** 2020-08-21

**Authors:** Igor Lyubutin, Sergey Starchikov, Ivan Troyan, Yulia Nikiforova, Marianna Lyubutina, Alexander Gavriliuk

**Affiliations:** 1 Shubnikov Institute of Crystallography of FSRC “Crystallography and Photonics” of Russian Academy of Sciences, 119333 Moscow, Russia; lyubutinig@mail.ru (I.L.); itrojan@mail.ru (I.T.); juliadavudova@gmail.com (Y.N.); lyuanne@mail.ru (M.L.); gavriliuk@mail.ru (A.G.); 2 Institute for Nuclear Research, Russian Academy of Sciences, Troitsk, 142190 Moscow, Russia

**Keywords:** high pressure, magnetically induced multiferroic, Mössbauer spectroscopy, phase transitions, synchrotron X-ray diffraction, diamond anvil cell, spin crossover, structural transitions, high-pressure effects in solids

## Abstract

Recently, the iron containing langasite-type crystal Ba_3_NbFe_3_Si_2_O_14_ has attracted great attention as a new magnetically induced multiferroic. In this work, magnetic, structural and electronic properties of the multiferroic Ba_3_NbFe_3_Si_2_O_14_ were investigated by several methods, including synchrotron X-ray diffraction, Raman spectroscopy and synchrotron Mössbauer source technique at high quasi-hydrostatic pressures (up to 70 GPa), created in diamond anvil cells. At room temperature, two structural transitions at pressures of about 3.0 and 17.5 GPa were detected. Mössbauer studies at high pressures revealed a radical change in the magnetic properties during structural transitions. At pressures above 18 GPa, the crystal transforms into two magnetic fractions, and in one of them the Néel temperature (*T_N_*) increases by about four times compared with the *T_N_* value in the initial phase (from 27 to 115 K). At pressures above 50 GPa, a spin crossover occurs when the fraction of iron Fe^3+^ ions in oxygen octahedra transits from the high-spin (*HS*, *S* = 5/2) to the low-spin (*LS*, *S* = 1/2) state. This leads to a new change in the magnetic properties. The magnetic ordering temperature of the *LS* sublattice was found to be of about 22(1) K, and magnetic correlations between *HS* and *LS* sublattices were studied.

## 1. Introduction

The langasite (La_3_Ga_5_SiO_14_) type crystals attract high scientific and practical interest because of observed high piezoelectric parameters, acousto-optic, and laser properties [1,2]. In addition, the “magnetic langasites” can be obtained by isomorphic introduction of magnetic 3*d* ions into the structure [3,4]. Recently, an iron containing langasite-type crystal Ba_3_NbFe_3_Si_2_O_14_ (BNFS) has attracted a great attention as a new magnetically induced multiferroic [3,4,5,6,7,8,9].

**Figure 1:**
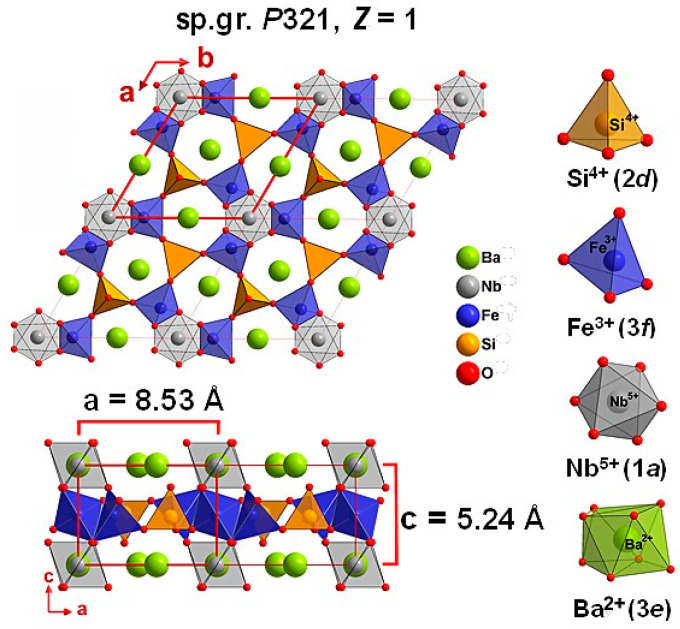
Crystal structure of Ba_3_NbFe_3_Si_2_O_14_ at ambient pressure and room temperature. The values of unit cell parameters are indicated.

At ambient conditions, BNFS has a trigonal crystal structure (space group *P*321, point group 32, Z = 1), where layers of oxygen tetrahedrons with Fe(3*f*) and Si(2*d*) ions alternate with the layers containing Nb(1*a*) and Ba(3*e*) ions, which are located in oxygen octahedrons and Thompson cubes, respectively [1,10] (Figure 1).

Mössbauer spectroscopy studies of magnetic properties revealed that magnetic moments of iron ions in BNFS arrange in three-dimensional antiferromagnetic order with the Neel temperature *T*_N_ of 27.1 K [3]. As found by neutron diffraction [11], below *T_N_*, Fe ions in 3*f* sites form a net of triangle magnetic clusters in the (*ab*) planes with a 120° orientation of iron magnetic moments, which appear due to frustrated exchange interactions (Figure 1). In addition, magnetic triangles of Fe moments form a helical arrangement along the *c* axis rotating from plane to plane with a period of about 7 unit cells [11,12]. Recently, the relationship between structural and magnetic chirality has been studied in detail in [13,14]. It was shown in [13] that, in the absence of helical axes in a noncentrosymmetric Ba_3_NbFe_3_Si_2_O_14_ crystal (and in a similar Ba_3_TaFe_3_Si_2_O_14_ compound), the formation of the spiral electron density of Fe(3*f*) and O3(6*g*) atoms in neighboring cells can be responsible for the phenomenon of crystalline chirality. The direction of rotation of the spiral is determined by the orientation of the ellipsoids of atomic displacements and correlates with the sign of rotation of the plane of light polarization by the crystal. The helical magnetic structure in BNFS was also recently investigated by Mössbauer spectroscopy [12].

A theoretical description of the helical magnetic structure and the conditions for the existence of multiferroic properties in langasites are presented in [5,6]. The low-temperature magnetic transition causes a structural transition from the *P*321 phase to the *P*3 (or *C*2) polar phase, which favors the appearance of ferroelectricity [5,13].

To create materials of practical importance, an increase in the critical temperatures of magnetic and ferroelectric transitions (*T*_N_ and *T*_P_) is a key problem. Modification of the crystal structure using chemical engineering or/and external pressure is the most promising way. Actually, this can be done either by applying an external pressure or by cationic substitution, which can create “internal” chemical pressure.

The effect of pressure on magnetoelectric properties in magnetic materials with triangular lattices was recently considered in [15,16]. Additional interest to this topic arose after the discovery of a huge increase in the value of Neel temperature *T*_N_ (from 27 to 120 K) in the langasite family compound Ba_3_TaFe_3_Si_2_O_14_ under high pressure above 20 GPa [17].

Meanwhile, new magnetic properties may appear in iron oxide materials under applied high pressures above 50 GPa, where spin-crossover effects are expected [18]. In particular, the transition of Fe^3+^ and Fe^2+^ ions from the high spin to the low spin state was observed experimentally in a number of iron oxide compounds with perovskite-like and garnet crystal structures [18].

Preliminary data on the study of the crystal structure and Mössbauer spectra in BNFS under high pressure were presented in Ref. [19]. The interpretation of the structural data was not entirely correct, since it was not clear whether the lattice symmetry and the unit cell change after structural transitions. Based on the available powder data, we made one of the most probable assumptions about the transition of BNFS into the hexagonal phase after 17–18 GPa. To confirm this, structural studies were carried out on BNFS single crystals at high pressures [20]. It was found that at a pressure of 3.5 GPa, the structure of this crystal transforms from the *P*321 phase (Z = 1) into the intermediate polar *P*3 (*Z* = 3) phase; and at a pressure of 17.5 GPa, the second structural transition to the perovskite-like phase with the space group $P\bar{6}2m\;\left(Z=1\right)$ and hexagonal symmetry occurs, which was previously unknown.

In the present work, the structural data of BNFS polycrystals were processed based on the results obtained for single crystals. The full-profile analysis of powder diffraction patterns was carried out up to pressures of 60 GPa and the lattice parameters of the structures before and after phase transitions were refined. Previously, the full-profile analysis could not be carried out because the lattice symmetry and unit cell after phase transitions were unknown. Based on the new data, an equation of state for langasite BNFS was constructed up to pressures of 60 GPa.

In view of the search and study the magnetic and electronic properties of the langasite family compounds, in this work, the Ba_3_NbFe_3_Si_2_O_14_ multiferroic was investigated by synchrotron Mössbauer source (SMS) technique at high pressures (up to 70 GPa) created in diamond anvil cells. The high-pressure Raman spectroscopy studies were also performed to establish correlations between the structural and magnetic properties in these crystals.

## 2. Results and Analysis

### 2.1. X-Ray Diffraction

**Figure 2:**
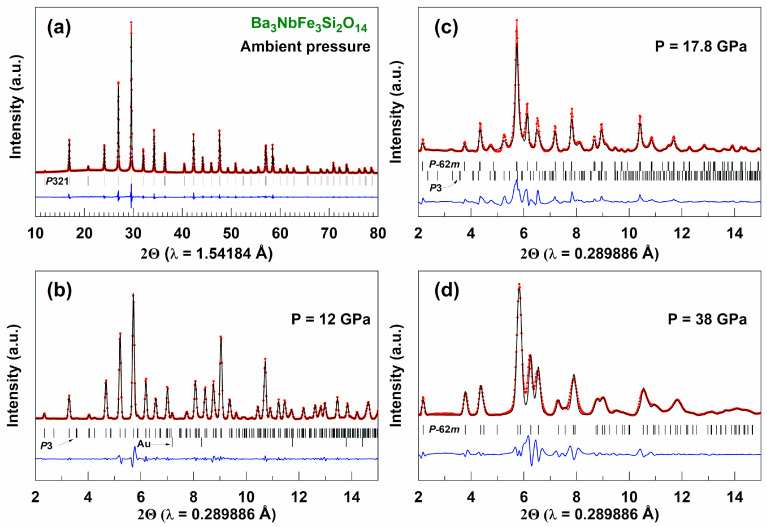
(Colour on-line) Representative powder XRD patterns of Ba_3_NbFe_3_Si_2_O_14_ showing the evolution of the crystal structure under applied pressure up to 58 Gpa: (**a**) Ambient pressure; (**b**) 12 GPa; (**c**) 17.8 GPa and (**d**) 38 GPa. Full-profile refinements of the structural modifications of BNFS are shown at selective pressures before and after phase transitions.

The phase composition of Ba_3_Nb^57^Fe_3_Si_2_O_14_ was controlled by X-ray powder diffraction and by the transmission Mössbauer spectroscopy. The X-ray diffraction pattern of BNFS shown in Figure 2a demonstrates a high quality compound, whose crystal structure belongs to the space group (sp. gr.) *P*321 with unit cell parameters *a* = 8.530(2) Å and *c* = 5.235(2) Å.

In applied pressure above 3 GPa, a set of diffraction peaks corresponding to a new phase with sp.gr. *P*3 was observed in the XRD patterns along with the peaks of the initial *P*321 phase. In a certain pressure range these two phases coexist, however above 12 GPa only the polar phase *P*3 remains (Figure 2b). With a further increase in pressure, the phase *P*3 persists up to 17.5 GPa, and then a new phase with sp. gr. $P\bar{6}2m$ appears (Figure 2c). The *P*3 and $P\bar{6}2m$ phases coexist in the pressure range of about 13–21 GPa, and then only the $P\bar{6}2m$ phase remains as the pressure further increases up to 58 GPa (Figure 2d).

Structural phase transitions at pressures of about 3.5 and 17–18 GPa are clearly detected, and this supports the data of single-crystal XRD studies of the BNFS compound performed recently in Ref. [20]. The effect of the coexistence of two phases in our BNFS sample, observed in the pressure region of structural transitions, can be explained by the powder nature of the sample and the pressure gradients at the sample in this experiment.

**Figure 3:**
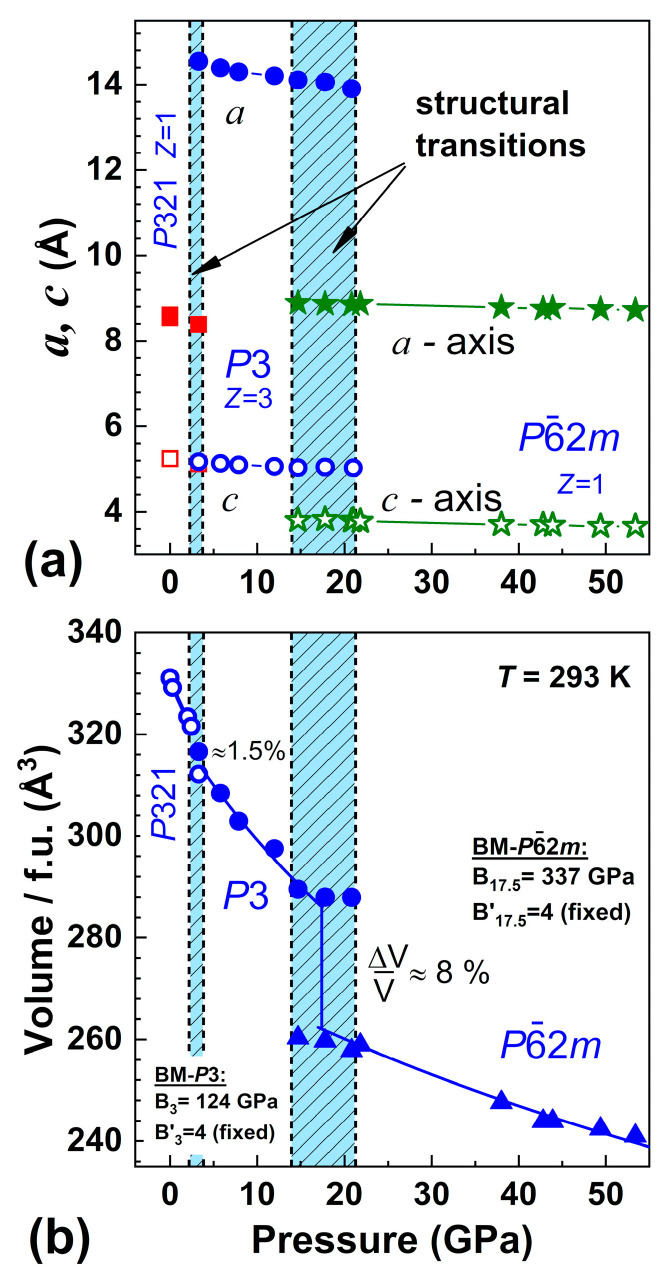
(Colour on-line) Pressure dependences of the unit cell parameters *a*, *c* (**a**) and the values of volume *V* for formula unit (**b**) in BNFS at room temperature. The shaded regions indicate the pressure interval of the coexistence of the structural phases. Symbols correspond to phases *P*321, *P*3, $P\bar{6}2m$.

Figure 3 shows the pressure behavior of the unit cell parameters *a* and *c* and the unit cell volume V. The second transition at 17.5 GPa leads to strong changes in the unit cell parameters and *a* sharp drop in the cell volume by about 8%. From the viewpoint of magnetic characteristics, an appreciable decrease in the parameter *c* during the structural transition at P ≈ 17.5 GPa should lead to a pronounced enhancement of the exchange coupling between iron ions located in neighboring (*ab*) planes.

### 2.2. Raman Spectroscopy 

**Figure 4:**
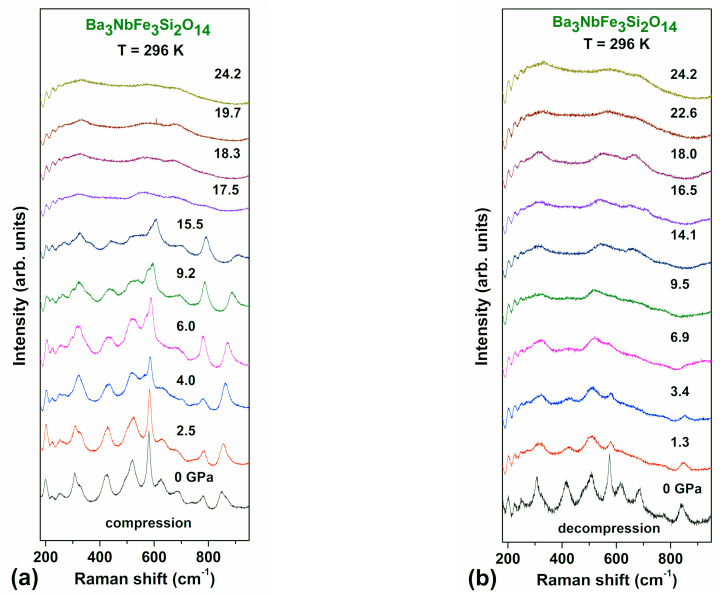
(Colour online) Raman spectra of Ba_3_NbFe_3_Si_2_O_14_ at different pressures up to *P* = 24 GPa at compression (**a**) and decompression (**b**).

Room temperature Raman spectra of Ba_3_Nb^57^Fe_3_Si_2_O_14_ at pressures up to 25 GPa for compression and decompression cycles are shown in Figure 4. The pressure behaviour of the positions of Raman peaks clearly demonstrates the appearance of two successive transitions under the compression run (Figure 4a). The first of them, at about 3–4 GPa, exhibits itself as a merger of some peaks, as well as in the appearance of a strong shoulder at 550 cm^−1^. This anomaly is consistent with the observation of a structural transition according to XRD and Mössbauer spectroscopy data. The second transformation occurs at about 17.5 GPa, where the Raman spectrum drastically changes. The sharp peaks completely disappear and only a few weak and very broad maxima are observed (Figure 4a). According to XRD, this is related to the second structural transition, which is accompanied by a decrease in the unit cell volume by about 8% (Figure 3).

**Figure 5:**
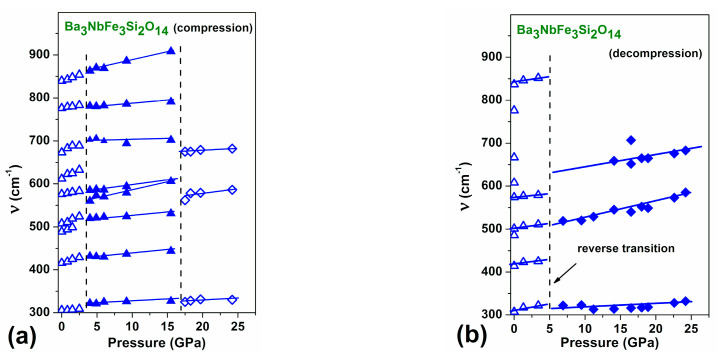
(Colour online) Pressure behavior of Raman peak positions in Ba_3_NbFe_3_Si_2_O_14_ at compression (**a**) and decompression (**b**). Dashed lines correspond to the structural transitions observed in XRD and Mössbauer experiments. Solid lines are guides to the eye.

The pressure behavior of the Raman peak positions (Figure 5) more clearly demonstrates the appearance of successive transitions at ~3 and 17.5 GPa. The transition at 17.5 GPa occurs abruptly and with a large hysteresis. 

During decompression, pronounced peaks appear only when the pressure is decreased to 3 GPa (Figure 4b). This demonstrates a very large hysteresis, which was also observed in the XRD measurements. This is indicative of a first order type of the structural phase transition at 17.5 GPa with a large energy barrier between the low- and high-pressure phases. When decompression to ambient pressure, the shape of the Raman spectrum returns to the initial sharp peaks characteristic of the low-pressure crystalline structure (Figure 4b and Figure 5b). This observation confirms the diffuse-like transition of the first order type with a strong reconstruction of the crystal structure. 

The results of the Raman experiment strongly support the existence of the first and second phase transitions and help locate more precisely the critical pressures of transitions.

### 2.3. High-Pressure Low-Temperature Mössbauer Spectroscopy

#### 2.3.1. Magnetic Properties of BNFS at Pressures in the Range 0–40 GPa

**Figure 6:**
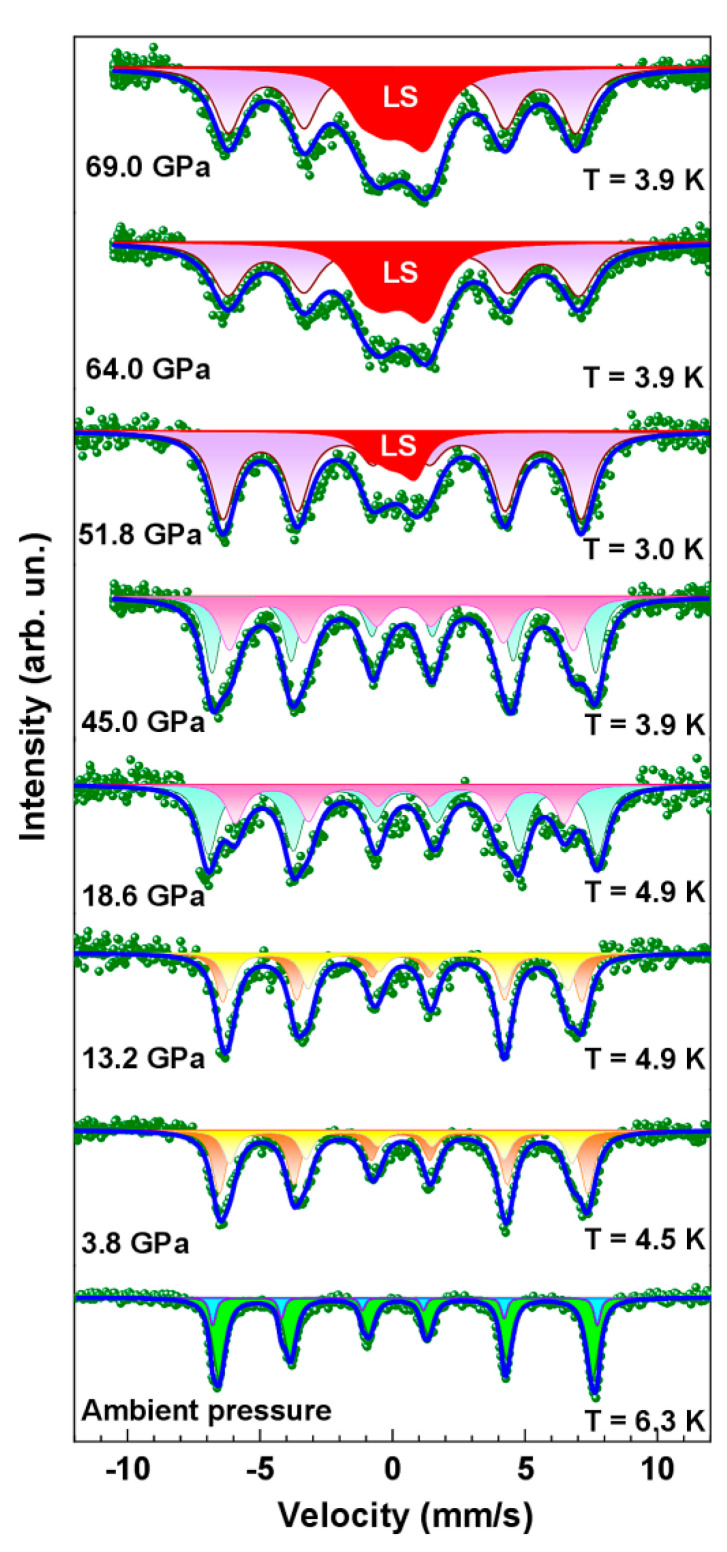
The low-temperature SMS spectra of Ba_3_NbFe_3_Si_2_O_14_ at different pressures up to *P* = 69 GPa. Solid lines are calculated subspectra fitted the experimental data. At pressures 51.8, 64.0 and 69.0 GPa, the LS sign indicates the Mössbauer subspectrum corresponding to iron ions in the low-spin state.

The magnetic and electronic properties of BNFS at high pressures up to 70 GPa and cryogenic temperatures were studied by synchrotron Mössbauer spectroscopy (SMS) technique. As shown in Figure 6, the SMS spectra of BNFS, recorded at the lowest temperatures in this experiment (3.0–4.9 K) are split into six main resonance lines demonstrating magnetic ordering of Fe ions at all pressures in the range from ambient to 45 GPa. 

As was established by neutron diffraction [11] and Mössbauer spectroscopy [12] studies, below *T_N_* and at ambient pressure, iron magnetic moments lying in the (*ab*) planes (in the 3*f* crystal sites) exhibit helical rotation with a propagation helicoidal vector along the *c* axis with a period of about 7*c* (*c* is the value of the unit cell parameter along the *c* axis) [11,12]. In this case, the Mössbauer spectrum, in general, should consist of a combination of 7 subspectra corresponding to the different orientation iron magnetic moment relative to the main axis of the electric field gradient (EFG) [12]. Obviously, the transformation of the spectra shapes under the applied pressure, shown in Figure 6, is related to the modification of the helical magnetic structure by pressure.

The average values of the hyperfine parameters obtained from the Mössbauer spectrum at ambient pressure and helium temperatures are: the isomer shift is δ = 0.345(5) mm/s and the magnetic hyperfine field at iron nuclei *H*_hf_ is about 45.77(5) T. These parameters correspond to the high spin state of Fe^3+^ ions (3*d*^5^, *S* = 5/2) in tetrahedral oxygen sites and imply essential covalence in Fe-O bonds. The value of the quadruple shift at 4.2 K is *ε* = −0.66 mm/s, which is in agreement with the quadrupole splitting of ∆ = 1.3 mm/s observed in the paramagnetic state at room temperature.

**Figure 7:**
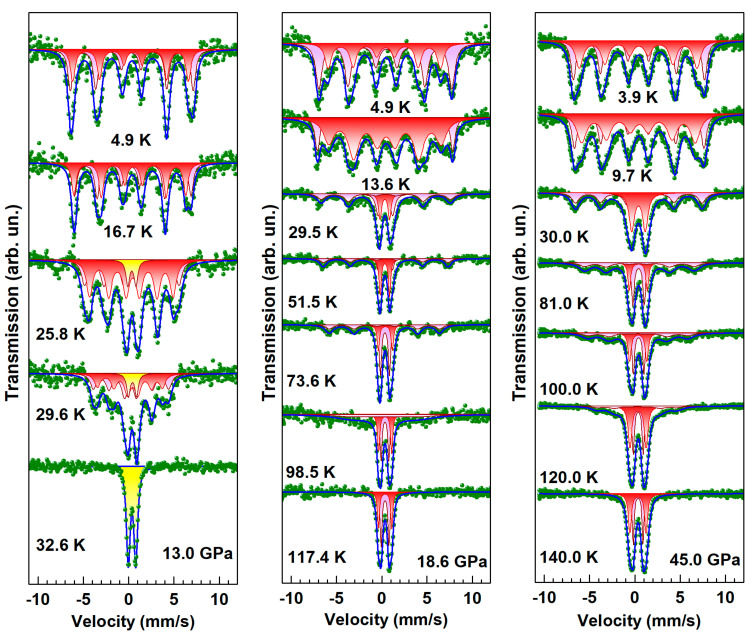
(Colour on-line) Evolution of SMS spectra with temperature in Ba_3_NbFe_3_Si_2_O_14_ at pressures below (13.0 GPa) and above (18.6 and 45.0 GPa) the structural transition at about *P* = 17.5 GPa. Color lines are the calculated subspectra fitted to the experimental points (dots).

**Figure 8:**
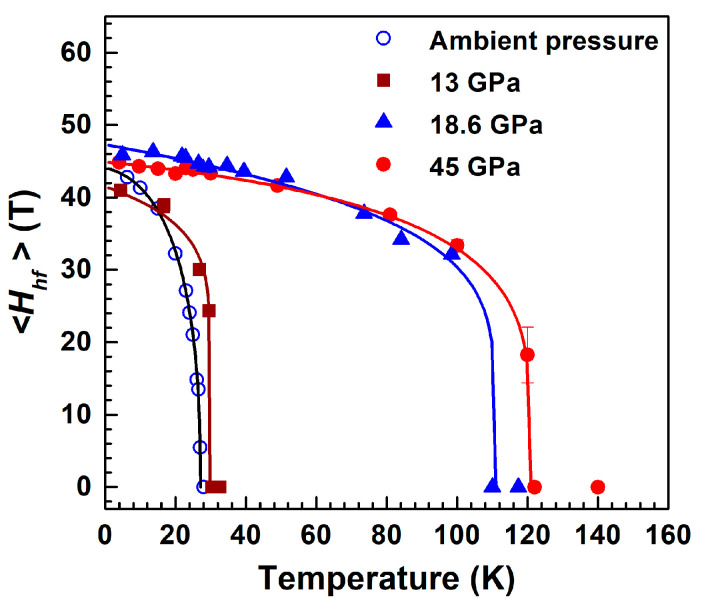
(Colour on-line) Temperature dependences of the average values of magnetic hyperfine field <*H*_hf_> at iron nuclei in the magnetic fraction of langasite Ba_3_NbFe_3_Si_2_O_14_ estimated from Mössbauer spectra at different pressures before and after the structural transitions. Solid lines are empirical function describing the pressure-temperature dependence of the field *H_hf_(P,T)* [17].

As temperature increases, the magnetic splitting of the Mössbauer spectra decreases, and the six-line magnetic spectra gradually transform into a quadrupole doublet indicating a transition to the paramagnetic state (Figure 7). The value of the Neel temperature *T*_N_ can be estimated from the temperature dependence of the magnetic hyperfine field at iron nuclei *H*_hf_ (Figure 8), and at ambient pressure the obtained value for BNFS is *T*_N_ = 27.2(1) K.

Under the applied pressure, the temperature evolution of the Mössbauer spectra does not changed essentially with increasing pressure from ambient to 17 GPa (Figure 7), while the *T*_N_ value slightly increases to about 32.0 K. However, at pressures above 18 GPa, the temperature evolution of the Mössbauer spectra change significantly (Figure 7). Along with the transition of the main fraction of the sample into the paramagnetic state at about 30 K, some part of the sample remains in magnetically ordered state even at rather high temperatures. This indicates the separation of the sample into two fractions with different magnetic properties. 

From the temperature dependence of the magnetic hyperfine field *H*_hf_ at, the temperature of the onset of magnetic ordering in a new magnetic phase can be estimated at about 110–115 K (Figure 8).

Obviously, the appearance of a new magnetic phase with high value of *T*_N_ is associated with a structural transition at pressure of 17.5 GPa, which was observed according to XRD and Raman spectroscopy studies. At this transition, Fe cations can change the local oxygen arrangement and/or redistribute between tetrahedral and octahedral oxygen sites.

At ambient conditions, iron ions in BNFS occupy 3*f* oxygen tetrahedra (Figure 1). The local nearest neighbors of Fe^3+^ are Si^4+^ and Nb^5+^, which occupy the tetrahedral 2*d* sites and octahedral 1*a* sites, respectively (Figure 1). At high pressures, redistribution of Fe^3+^ ions can occur either between two types of tetrahedral 3*f* and 2*d* sites in the (*ab*) plane or/and between tetrahedral 3*f* and octahedral 1*a* sites in the nearest layers along the *c* axis.

In particular, when iron ions partially occupy the 2*d* tetrahedral sites, replacing Si, a very strong magnetic interaction can appear between the Fe(3*f*) and Fe(2*d*) ions in the (*ab*) plane, since the Fe(3*f*)-O2(6*g*)-Fe(2*d*) bond distances and the bond angle are very effective for strong superexchange interaction [10,20]. In addition, under high pressure, Fe ions can partially be redistributed between tetrahedral 3*f* and octahedral 1*a* sites in the nearest layers along the *c* axis (Figure 1). This will initiate the Fe(3*f*)-O-Fe(1*a*) superexchange interaction. 

The high pressure XRD studies of BNFS revealed a drastic decrease in the lattice parameters *a* and *c* during the structural transition at a pressure of about 17.5 GPa (Figure 3). A decrease in the *c* parameter should substantially increase the exchange interaction between Fe ions in adjacent (*ab*) planes, leading to a further increase in the Neel point.

On the other hand, the applied pressure can initiate the displacement of oxygen atoms around the Fe ions. Our recent X-ray diffraction studies of the single crystal established two modifications of crystal structures of BNFS, which are stable in the pressure ranges of 3.5–17.0 GPa and 17.5–32.5 GPa [19]. At pressures above 3.2 GPa, BNFS undergoes a continuous phase transition from the *P*321 phase into an intermediate structure with space group *P*3. The creation of the polar *P*3 phase in BNFS leads to the appearance of ferroelectric properties, and such a crystal can be considered as the pressure-induced multiferroic.

**Figure 9:**
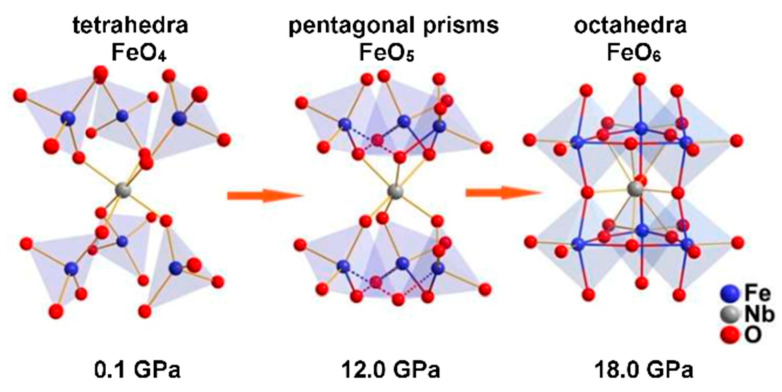
Structural fragments from Fe-O polyhedra of polymorph modifications of Ba_3_NbFe_3_Si_2_O_14_ stable at different pressures. The transformation of the Fe^3+^ coordination from tetrahedral to octahedral through the trigonal bipyramid under pressure is shown.

A smooth decrease in the Fe-O5 distance during this transition results in the addition of a fifth oxygen to the environment of the iron ion. Figure 9 shows fragments of Fe-O polyhedra of polymorphic modifications of BNFS at pressures of 0.1, 12.0 and 18.0 GPa. Small atomic displacements lead to an increase in the iron coordination number from FeO_4_ to FeO_5_ (Figure 9). 

The second structural transition at a pressure of about 17.5 GPa leads to the transformation of the crystalline structure with an increase in symmetry to the sp. gr. $P\bar{6}2m$ (Figure 2d). This new modification can be considered as a perovskite-like structure [19]. In this case, a direct bond Fe-O-Fe is formed between Fe atoms in the nearest (*ab*) planes, and each Fe atom in the structure acquires the octahedral coordination of oxygen atoms (Figure 9). The appearance of new direct Fe-O bonds leads to a strong increase in the values of the superexchange interactions between Fe^3+^ cations that can result in growing of the Neel temperature

#### 2.3.2. Spin Crossover and Magnetic Properties of BNFS at Pressures above 45 GPa

**Figure 10:**
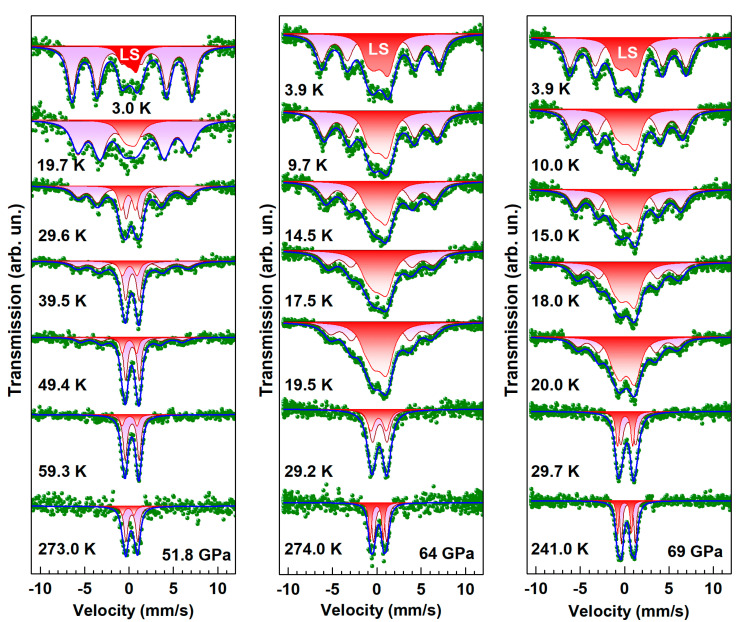
(Colour on-line) Evolution of SMS spectra with temperature in Ba_3_NbFe_3_Si_2_O_14_ at pressure of 51.8, 64.0 and 69.0 GPa. Color lines are the calculated subspectra fitted to the experimental points (dots). The LS sign indicates the Mössbauer subspectrum corresponding to iron ions in the low-spin state.

The appearance of fragments of a crystalline structure with iron ions in the octahedral oxygen sites was further supported by the Mössbauer spectroscopy studies of BFNS at pressures above 45 GPa. Figure 10 demonstrates the temperature evolution of the Mössbauer SMS spectra at pressures of 52, 64 and 69 GPa, which are above the pressure of the third transition observed in BFNS at about 42 GPa by optical absorption and XRD studies [20,21].

At the lowest temperature of 3.0–3.9 K, the SMS spectra of BNFS consist of two sets of the magnetically split lines with different values of the magnetic hyperfine fields *H*_hf_ at the iron nuclei. The first magnetic component with *H*_hf_ = 42 T is typical of Fe^3+^ ions in the high-spin state (3*d*^5^, *HS*, *S* = 5/2) in tetrahedral oxygen sites as was observed at ambient and low pressures in the BNFS sample. The second magnetic component with the *H*_hf_ value of about 8–9 T corresponds to the low-spin Fe^3+^ ions (*LS*, *S* = 1/2).

As was shown in Ref. [17,22], a low spin state of Fe^3+^ ions cannot be created in the tetrahedral oxygen sites at such pressures, since the energy of the *LS* term is much higher than the energy of the *HS* term [22]. A theoretical consideration of the possible spin crossover *HS* (*S* = 5/2) → *LS* (*S* = 1/2) induced by high pressure in a similar langasite compound Ba_3_TaFe_3_Si_2_O_14_ [17] predicted a much higher pressure value of the spin-crossover transition for the tetrahedral iron sites (about 800 GPa) compared with the octahedral iron sites (about 70 GPa).

**Figure 11:**
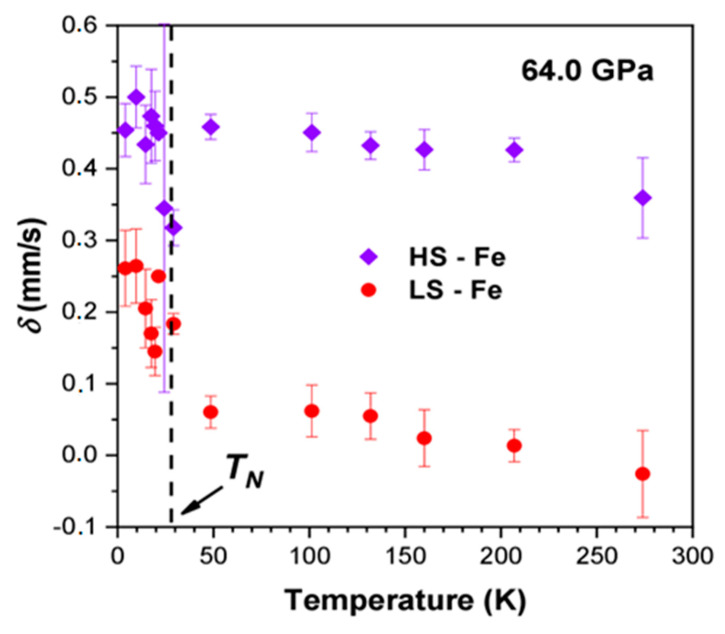
(Colour on-line) Temperature dependences of isomer shift values for *HS* and *LS* fractions of iron ions in Ba_3_NbFe_3_Si_2_O_14_ obtained from Mössbauer spectra at a pressure of 64.0 GPa. The vertical dashed line corresponds to the point of magnetic collapse (Neel temperature).

Thus, we can conclude that the magnetic component with a low value of *H*_hf_ (typical of the *LS* state of Fe^3+^) corresponds to iron ions in the octahedral sites of BNFS in the high-pressure phase. Further support of the occurrence of the *LS* state of Fe^3+^ follows from the values of the isomer shift *δ* in the Mössbauer spectra at high pressures (Figure 11). The *δ* value of the *LS* phase is temperature dependent and decreases from about 0.2 to 0.0 mm/s with increasing temperature from 3.5 to 295 K (Figure 11). Meanwhile, the δ value of the *HS* phase is about 0.42–0.45 mm/s at the lowest temperature, and only slightly decreases with a temperature rise in accordance with the behavior of the second order Doppler shift.

In addition, a very high value of the quadrupole splitting parameter Δ of about 1.7 mm/s, revealed in Mössbauer spectra at *P* > 50 GPa, is a characteristic of the *LS* state of Fe^3+^ ions. Moreover, the reduction of the optical gap *Eg* observed in BNFS down to a value of 0.7 eV, typical of semiconductors, is also a signature of the *HS-LS* crossover in iron oxides [21].

## 3. Discussion

Now, several very interesting conclusions about the magnetic behaviour of this compound can be obtained from the temperature evolution of the hyperfine parameters of the Mössbauer spectra. 

1. First of all, from the area of the Mössbauer lines we can estimate the volume fraction of iron ions in the *LS* and *HS* states. In fact, this corresponds to the fraction of octahedral and tetrahedral sites occupied by iron ions in the high-pressure phases of BNFS at *P* > 52 GPa. Below, we will call them *HS* and *LS* sublattices. 

2. From the temperature dependence of the magnetic hyperfine field *H*_hf_, the values of magnetic ordering temperatures (Neel points) for iron ions in the high-spin and low-spin states can be obtained.

3. Specific magnetic properties of the material, which are due to interactions between iron ions in the *HS* (tetrahedral) and *LS* (octahedral) sublattices can be discovered.

**Figure 12:**
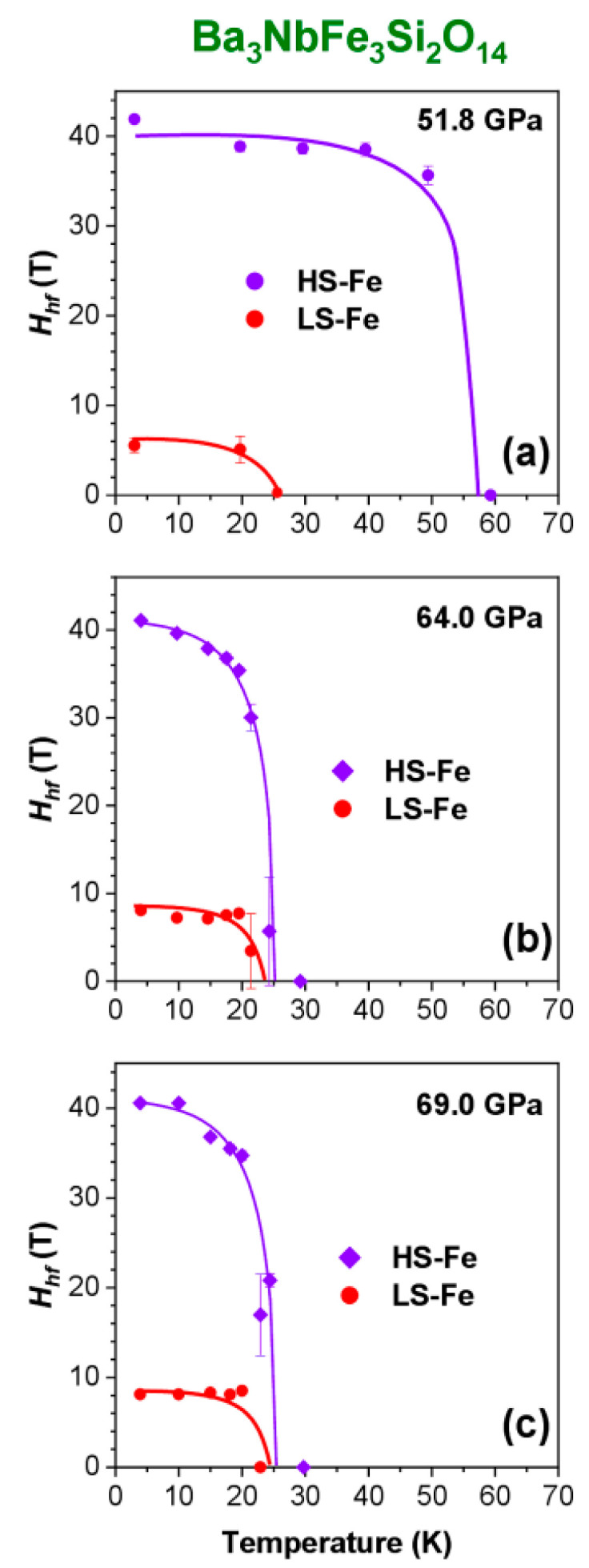
(Colour on-line) Temperature dependences of the magnetic hyperfine field *H*_hf_ at iron nuclei in Ba_3_NbFe_3_Si_2_O_14_ estimated from Mössbauer spectra at high pressures above the spin crossover *HS* → *LS* transition. (**a**) 51.8 GPa; (**b**) 64 GPa and (**c**) 69 GPa. Solid lines are the guides for the eye.

Figure 12 shows the temperature dependence of the magnetic fields *H*_hf_ in a BNFS sample at pressure of 51.8, 64.0 and 69.0 GPa. At highest pressures (64.0 and 69.0 GPa), the field *H*_hf_ in the *HS* sublattice gradually decreases from about 41 T to zero with increasing temperature from 3.5 to 22.0 K. In the *LS* sublattice, the field *H*_hf_ value is practically independent of temperature, remaining at the level of about 8.5 T, and drastically drops to zero at about 22.0 K (Figure 12). Remarkably, that both *HS* and *LS* sublattices transit to a paramagnetic state at the same temperature, which implies a magnetic coupling and exchange interaction between these sublattices. Obviously, the magnetic transition temperature can be considered as the Neel point of the *LS* sublattice *T*_N_ = (22.0 ± 1.0) K. 

Since the hyperfine field *H*_hf_ is directly proportional to the iron magnetic moment, the temperature behaviour of *H*_hf_ in the *LS* sublattices is characteristic of a low-dimensional Ising-type magnetic system.

As follows from the area of Mössbauer resonance lines, the fractions of the *HS* and *LS* components at pressures 64 and 69 GPa are in a ratio of about 60/40. However, at a pressure of 51.8 GPa this ratio is about 80/20. This may indicate that not all Fe ions in octahedral sites transit to the *LS* state at this pressure, which may be due to the pressure gradient in the high-pressure cell.

Meanwhile, the magnetic properties of the BNFS sample at *P* = 51.8 GPa differ from those at pressures of 64 and 69 GPa. At higher pressures (64 and 69 GPa), the magnetic ordering in the *HS* and *LS* sublattices is simultaneously collapsed at the same temperature of about 22 K.

On the other hand, at a pressure of 51.8 GPa, the magnetic correlations in the *LS* sublattice disappear at a temperature of about 20 K, whereas, the *HS* sublattice transforms to the paramagnetic state at a much higher temperature of about 58 K (Figure 12). Most probably, at a pressure of 51.8 GPa, not all Fe ions in the octahedral sites pass to the *LS* state (only half of them: compare 40% and 20%). The remaining part of the *HS* iron ions in the octahedral sites interacts with the *HS* iron ions in the tetrahedral sites leading to a relatively high magnetic ordering temperature of about 58 K.

As shown above, at pressures in the range 18–45 GPa, when all the Fe ions in the octahedral sites were in the *HS* state, the Neel temperature of the *HS* sublattice was about 115 K (Figure 8). At 51.8 GPa, when half of the octahedral *HS* Fe ions transferred to the *LS* state, the Neel temperature decreases from 115 to 58 K. 

This analysis shows that the pressure value of 52 GPa can be considered as the onset point of the *HS* → *LS* transition in the Fe octahedral sites of the high-pressure phase of BNFS. This value is very similar to that found previously in other iron oxides with garnet (YIG), perovskite-like and orthorhombic crystal structures in such compounds as RFeO_3_, BiFeO_3_, Fe_2_O_3_, FeBO_3_ [18]. The magnetic correlation and the interaction between the *LS*-octahedral and *HS*-tetrahedral sublattices in BNFS are similar to the behavior of two iron sublattices in yttrium iron garnet Y_3_Fe_5_O_12_ [23]. In this garnet Fe^3+^ ions occupy both octahedral [a] and tetrahedral (d) sites in a ratio of 40/60, that is {Y_3_}[Fe_2_](Fe_3_)O_12_. As was observed in Ref. [23], under a pressure of about 50 GPa, the *HS* → *LS* crossover occurs in Fe ions of the octahedral sites of the garnet, leading to magnetic collapse in both sublattices. 

The ratio of *LS/HS* = 40/60 in our langasite BNFS is the same as the ratio of occupation of [a] and (d) sites in the garnet. It can be supposed that some clusters with a garnet-like structure appear in BNFS at pressures above 42 GPa, where the structural and optical anomaly was detected by X-ray diffraction and optical absorption measurements [21]. The character of the magnetic interaction and the exchange coupling between the octahedral and tetrahedral sublattices in this langasite Ba_3_NbFe_3_Si_2_O_14_ at pressures above 52 GPa is similar to the behavior of garnet Y_3_Fe_5_O_12_ at high pressure.

**Figure 13:**
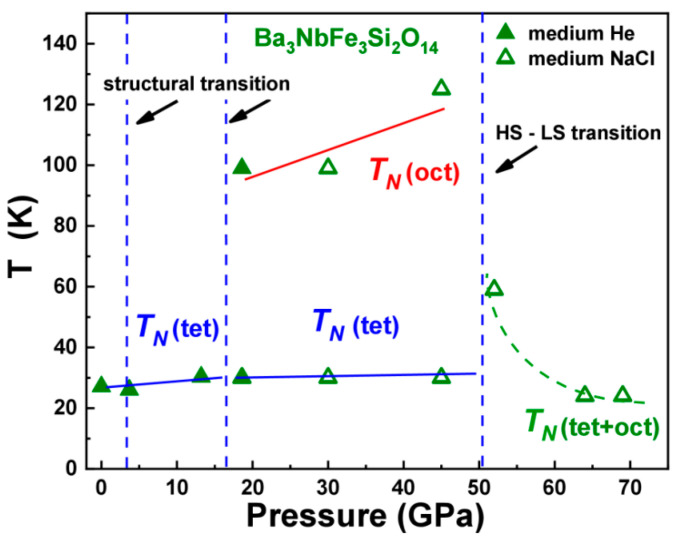
The magnetic *P-T* phase diagram of langasite Ba_3_NbFe_3_Si_2_O_14_ demonstrating an occurrence of three magnetic phases with different values of *T*_N_ in different intervals of pressure. Solid lines correspond to the Neel temperatures and separate magnetic states of iron in tetrahedral and octahedral fractions of the sample. A dashed green line corresponds to the magnetic behavior of the compound in the low-spin state. Vertical dashed lines separate the different crystal and electronic phases.

Based on the structural, magnetic and electronic properties of Ba_3_NbFe_3_Si_2_O_14_ obtained, a magnetic phase *P-T* diagram was constructed (Figure 13). It demonstrates the P-T regions of different magnetic and electronic phases and their correlation with the crystal structure at extreme conditions of high pressures and low temperatures.

## 4. Materials and Methods

Polycrystalline samples of Ba_3_NbFe_3_Si_2_O_14_ were synthesized by a ceramic synthesis technology from precursor oxides and salts [9]. For Mössbauer studies, the iron used in the sample synthesis was enriched with ^57^Fe isotope to 50%.

At ambient conditions, powder X-ray diffraction data of Ba_3_Nb^57^Fe_3_Si_2_O_14_ were obtained with Rigaku Miniflex-600 diffractometer (Rigaku Corporation, Tokyo, Japan) using Cu-*Kα* radiation (40 kV, 15 mA, Ni-*Kβ* filter) in the 2*θ* range 10–80° at a scan speed 1 °/min.

High pressure experiments were performed with diamond anvil cells (DAC). Polycrystalline compact samples were squeezed between diamonds and placed in the working volume of the rhenium gasket in the DAC. The cullet diameter of diamond anvils was about 200 μm, and the hole diameter in the gasket was about 80 μm. Helium and NaCl were used as the quasihydrostatic pressure-transmitting medium. The pressure was measured by the shift of the ruby fluorescence line [24] Several ruby chips were placed at different distances from the center of the gasket hole to evaluate the pressure gradient in the cell.

X-ray diffraction patterns of Ba_3_Nb^57^Fe_3_Si_2_O_14_ at high pressures up to 55 GPa were recorded using P02.2 Extreme Condition Beamline [25] (DESY, PETRA III, Hamburg, Germany). The synchrotron radiation with the wavelength λ = 0.289886 Å and spot size on the sample of 2 × 2.2 µm in DAC was used. The two-dimensional Perkin Elmer (XRD 1621) detector was set at a distance of 462.92 mm from the sample. The refinement of the unit cell parameters of the high-pressure phases was carried out as a result of a full-profile analysis by the Le Bail method [26] in the JANA2006 software [27].

Raman spectra at high pressures up to 25 GPa were obtained at room temperature using a Princeton Instruments Acton SP2500 monochromator/spectrograph (Teledyne Princeton Instruments, Trenton, NJ, USA) equipped with Spec-10 system with a nitrogen-cooled CCD detector. A krypton laser with a 647.1 nm line was used as an excitation source for the Raman signal. 

The magnetic and electronic properties of BNFS at high pressures up to 70 GPa and in the temperature range from 3.0 to 295 K for each pressure point were studied by the synchrotron Mössbauer source technique (SMS) [28,29] in the European Synchrotron Radiation Facility Center (ESRF, Grenoble, France). Mössbauer absorption spectra were measured at the Nuclear Resonance beamline ID18. For the model processing of Mössbauer spectra, we used the MossA program specially developed for the analysis of synchrotron Mössbauer spectra (the MossA software package [30]). When approximating the spectra, the total transmission integral was calculated. The source line was approximated by the squared Lorentzian distribution, taking into account the peculiarities of synchrotron Mössbauer radiation [28], and for the absorber line this was done by the conventional Lorentzian one. The source line width was estimated from an experiment with a thin standard absorber of K_2_Mg^57^Fe(CN)_6_ with a single line of known width. During the experiment, the source linewidth was monitored and evaluated before and after each measurement of the Mössbauer spectrum on the BNFS sample. The model used the following constraints:

(1) At pressures above 50 GPa, for temperatures T < T_N_, the line width (FWHM) for the LS and HS magnetically split components was assumed to be the same and varied.

(2) To a first approximation, in six-line Mössbauer spectra, the ratio of the intensities of the lines (1st,6th): (2nd,5th): (3rd,4th) was considered characteristic of a powder sample 3: 2: 1 for both HS and LS sextets and then varied. When varied, the ratio of the intensities of the 1st: 2nd: 3rd: 4th: 5th: 6th lines in the HS and LS sextets looks like 3: x: 1: 1: x: 3, where x varies during fitting in the range 0–4.

(3) Based on the fit of the spectrum at the lowest temperature of 3 K, the ratio of fractions of the HS and LS components was fixed in the entire temperature range. At pressures 64 and 69 GPa, it was HS: LS = 60:40, and at 51 GPa it was HS: LS = 80:20. At temperatures above T_N_, the spectra were fitted with two doublets (for the HS and LS components) with the corresponding area ratios.

**Figure 14:**
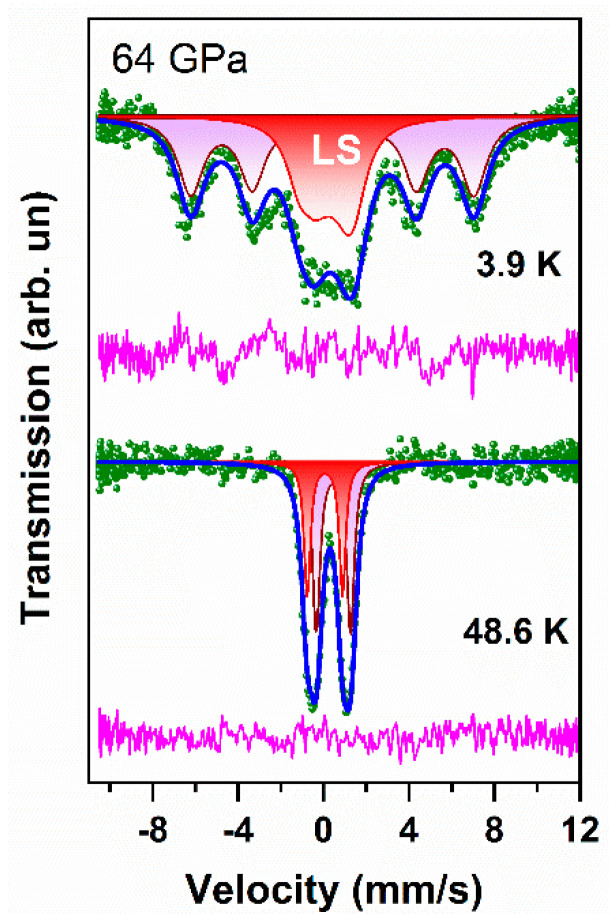
Mössbauer spectra of BNFS at temperatures 3.9 and 48 K shown for comparison in the same scale. Obviousely, the line width in the doublets (at 48 K) is much smaller than the width of the magnetic spectrum at 3.9 K.

For comparison, Figure 14 shows the BNFS Mössbauer spectra at 3.9 and 48 K for comparison on the same scale. It is clearly seen that the line width in doublets (at 48 K) is much less than the width of the magnetic spectrum at 3.9 K. Obviously, in terms of the width and asymmetry of the resonance lines, the magnetic sextet is much more suitable for fitting experimental spectra than the paramagnetic doublet. This indicates the magnetic nature of the line broadening. 

## 5. Conclusions

It is shown that in the langasite family Ba_3_NbFe_3_Si_2_O_14_ compound, external pressure strongly affects the crystal structure and leads to structural, magnetic and electronic transitions. At pressure above 3.2 GPa, BNFS undergoes a phase transition from the *P*321 phase into intermediate structure with space group *P*3. The creation of the polar *P*3 phase leads to the appearance of ferroelectric properties, and this crystal can be considered as a pressure-induced multiferroic. During the *P*321 → *P*3 transition the oxygen coordination of Fe ions gradually changes from FeO_4_ to FeO_5_.

At pressures above 18 GPa, part of the iron ions passes from tetrahedral coordination to octahedral FeO_6_, and fragments with a perovskite-like structure with sp. gr. $P\bar{6}2m$ appear. This entails an enhancement of exchange interactions, and the temperature of magnetic ordering in the octahedral Fe sites increases from about 30 K to 115 K. 

At pressures above 45 GPa, further transformation of the structure occurs, and fragments with the coexisting FeO_4_ and FeO_6_ sites appear. The magnetic interaction between iron ions in these sites resembles the behavior of the octahedral and tetrahedral iron sublattices in the yttrium-iron garnet YIG. At pressures above 52 GPa, a spin crossover occurs in the Fe^3+^ ions in the octahedral sites of BNFS and iron ions transit from the high-spin state *S* = 5/2 to the low-spin state *S* = 1/2. This causes a decrease in the value of the Néel point. The estimated value of the magnetic ordering temperature (*T*_N_ point) for the *LS* phase of BNFS is about 22.0 ± 1.0 K. At higher pressures the magnetic ordering in the *HS* and *LS* sublattices collapses simultaneously at one temperature of about 22 K, which indicates a strong coupling between the FeO_4_ and FeO_6_ sites.
